# 2,2-Diphenyl-*N*-{[2-(tri­fluoro­meth­yl)phen­yl]carbamo­thio­yl}acetamide

**DOI:** 10.1107/S1600536813018680

**Published:** 2013-07-13

**Authors:** Mohd Sukeri Mohd Yusof, Nur Rafikah Razali, Suhana Arshad, Azhar Abdul Rahman, Ibrahim Abdul Razak

**Affiliations:** a Department of Chemical Sciences, Faculty of Science and Technology, Universiti Malaysia Terengganu, Mengabang Telipot, 21030 Kuala Terengganu, Malaysia; b School of Physics, Universiti Sains Malaysia, 11800 USM, Penang, Malaysia

## Abstract

The title mol­ecule, C_22_H_17_F_3_N_2_OS, adopts a *trans*–*cis* conformation with respect to the positions of the carbonyl and tri­fluoro­methyl­benzene groups against the thio­carbonyl group across the C—N bonds. The mol­ecular structure is stabilized by an intra­molecular N—H⋯O hydrogen bond with an *S*(6) ring motif. The tri­fluoro­methyl-substituted benzene ring forms dihedral angles of 66.05 (9) and 47.19 (9)° with the terminal phenyl rings and is twisted from the O=C—N—(C=S)—N carbonyl­thio­urea plane [maximum deviation = 0.0535 (12) Å], making a dihedral angle of 63.59 (8)°. In the crystal, N—H⋯O and C—H⋯F hydrogen bonds link the mol­ecules into a layer parallel to the *bc* plane. A C—H⋯π inter­action is also observed.

## Related literature

For the biological activity of thio­urea derivatives, see: Vankatachalam *et al.* (2001 ▶). For related structures, see: Yusof, Arshad *et al.* (2012 ▶); Yusof, Embong *et al.* (2012 ▶); Yusof, Mutalib *et al.* (2012 ▶). For hydrogen-bond motifs, see: Bernstein *et al.* (1995 ▶). For bond-length data, see: Allen *et al.* (1987 ▶). For the stability of the temperature controller used for the data collection, see: Cosier & Glazer (1986 ▶).
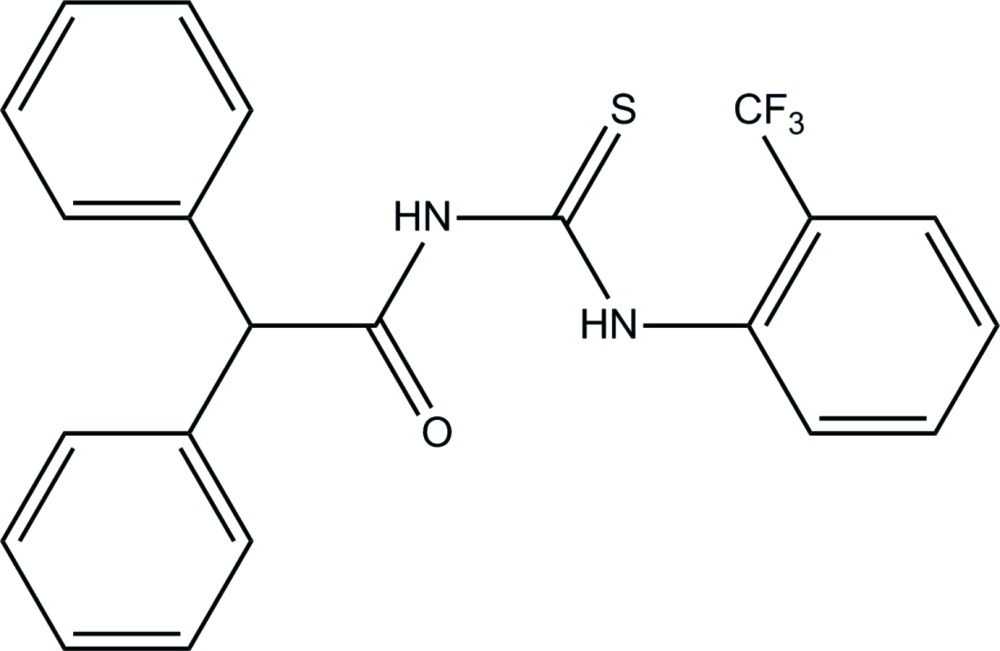


## Experimental

### 

#### Crystal data

C_22_H_17_F_3_N_2_OS
*M* *_r_* = 414.44
Orthorhombic, 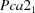

*a* = 20.0318 (4) Å
*b* = 10.2866 (2) Å
*c* = 9.5351 (2) Å
*V* = 1964.79 (7) Å^3^
*Z* = 4
Mo *K*α radiation
μ = 0.21 mm^−1^
*T* = 100 K
0.56 × 0.18 × 0.06 mm

#### Data collection

Bruker SMART APEXII CCD area-detector diffractometer
Absorption correction: multi-scan (*SADABS*; Bruker, 2009 ▶) *T*_min_ = 0.892, *T*_max_ = 0.987
21265 measured reflections
5618 independent reflections
4608 reflections with *I* > 2σ(*I*)
*R*_int_ = 0.047

#### Refinement

*R*[*F* ^2^ > 2σ(*F* ^2^)] = 0.044
*wR*(*F* ^2^) = 0.081
*S* = 1.02
5618 reflections
270 parameters
2 restraints
H atoms treated by a mixture of independent and constrained refinement
Δρ_max_ = 0.24 e Å^−3^
Δρ_min_ = −0.25 e Å^−3^
Absolute structure: Flack (1983 ▶), 2568 Freidel pairs
Flack parameter: 0.01 (6)

### 

Data collection: *APEX2* (Bruker, 2009 ▶); cell refinement: *SAINT* (Bruker, 2009 ▶); data reduction: *SAINT*; program(s) used to solve structure: *SHELXTL* (Sheldrick, 2008 ▶); program(s) used to refine structure: *SHELXTL*; molecular graphics: *SHELXTL*; software used to prepare material for publication: *SHELXTL* and *PLATON* (Spek, 2009 ▶).

## Supplementary Material

Crystal structure: contains datablock(s) global, I. DOI: 10.1107/S1600536813018680/is5283sup1.cif

Structure factors: contains datablock(s) I. DOI: 10.1107/S1600536813018680/is5283Isup2.hkl

Supplementary material file. DOI: 10.1107/S1600536813018680/is5283Isup3.cml

Additional supplementary materials:  crystallographic information; 3D view; checkCIF report

## Figures and Tables

**Table 1:** Hydrogen-bond geometry (Å, °) *Cg*1 is the centroid of the C1–C6 ring.

*D*—H⋯*A*	*D*—H	H⋯*A*	*D*⋯*A*	*D*—H⋯*A*
N1—H1*N*1⋯O1	0.96 (3)	1.93 (2)	2.6237 (19)	127 (2)
N2—H1*N*2⋯O1^i^	0.81 (2)	2.04 (2)	2.838 (2)	174 (2)
C9—H9*A*⋯F1^ii^	0.95	2.53	3.395 (2)	151
C7—H7*A*⋯*Cg*1^iii^	1.00	2.84	3.7826 (19)	158

Symmetry codes: (i) 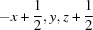; (ii) 
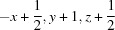
; (iii) 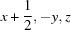.

**Fig. 1.**
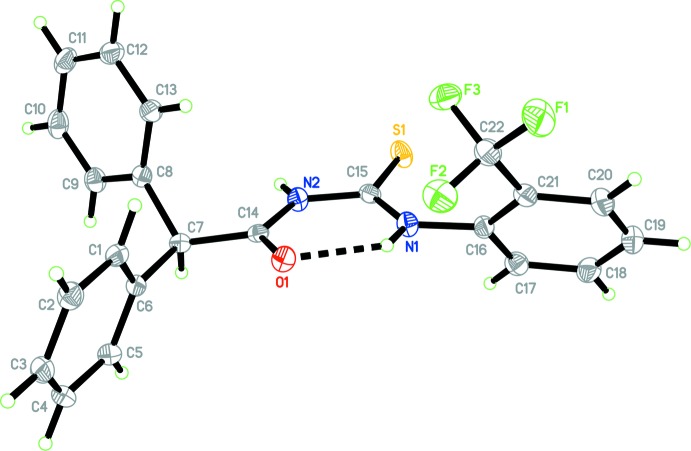
The molecular structure of the title compound with atom labels with 50% probability displacement ellipsoids. The dashed line represents the intramolecular hydrogen bond.

**Fig. 2.**
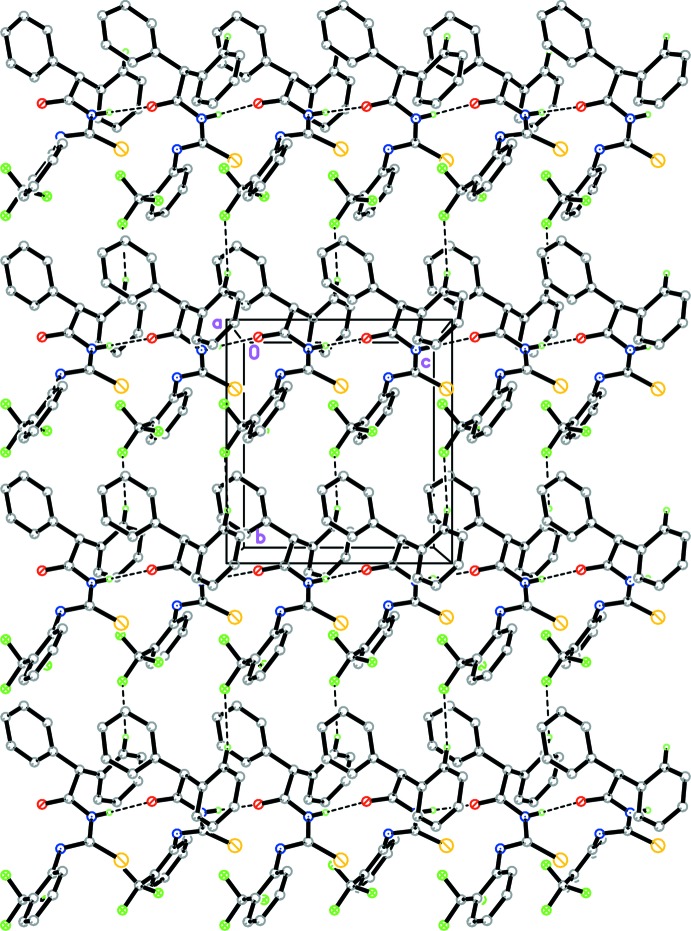
The crystal packing of the title compound. The H atoms not involved in the intermolecular interactions (dashed lines) have been omitted for clarity.

## supplementary crystallographic information

### Comment

Recent studies have shown that thiourea derivatives are potential biologically active agents, such as antimicrobials and HIV inhibitors (Vankatachalam *et al.*, 2001). The molecular structure of the title compound is shown in Fig. 1. The bond lengths (Allen *et al.*, 1987) and angles are within normal ranges and are comparable to the related structures (Yusof, Arshad *et al.*, 2012; Yusof, Embong *et al.*, 2012; Yusof, Mutalib *et al.*, 2012). The molecule adopts a *trans*-*cis* configuration with respect to the positions of diphenylmethane and trifluoromethylbenzene (F1–F3/C16–C22) groups, respectively, to the sulfur (S1) atom across the C—N bond. The trifluoromethyl-substituted benzene ring (C16–C21) forms dihedral angles of 66.05 (9) and 47.19 (9)° with the terminal phenyl rings, C1–C6 and C8–C13, respectively. Furthermore, the trifluoromethylbenzene plane (C16–C22) is slightly twisted from the carbonyl thiourea moiety (S1/O1/N1/N2/C15/C14) with a C15—N1—C16—C21 torsion angle of 119.3 (2)°. In the molecule, an intramolecular N2—H1N2···O1 hydrogen bond forms an *S*(6) graph-set motif (Bernstein *et al.*, 1995).

In the crystal (Fig. 2), molecules are linked into a one-dimensional chain along the *c*-axis *via* intermolecular N2—H1N2···O1 hydrogen bonds (Table 1) and further connected into a two dimensional layer parallel to the *bc*-plane by intermolecular C9—H9A···F1 hydrogen bonds (Table 1). In addition, a C7—H7A···*Cg*1 (Table 1) interaction is also observed in the crystal structure (*Cg*1 is the centroid of C1–C6).

### Experimental

An acetone (30 ml) solution of 2-triflouroaniline (1.25 g, 8.4 mmol) was added to a round-bottom flask containing 2,2-diphenylacetyl chloride (1.93 g, 8.4 mmol) and ammonium thiocyanate (0.64 g, 8.4 mmol). The mixture was put at reflux for 1.5 H then filtered off and left to evaporate at room temperature. The colourless precipitate obtained was washed with water and cold ethanol. Colourless crystals suitable for X-ray analysis were obtained by recrystallization of the precipitate in acetone.

### Refinement

N-bound H atoms were located in a difference Fourier map. Atom H1N1 was refined freely [N—H = 0.96 (3) Å], while atom H1N2 was refined with a bond restraint N—H = 0.85 (2) Å [refined distance: N1—H1N1 = 0.807 (15) Å]. The remaining H atoms were positioned geometrically (C—H = 0.95 or 1.00 Å) and refined using a riding model with *U*_iso_(H) = 1.2*U*_eq_(C). In the final refinement, one outlier was omitted (10 0 -7).

### Crystal data

**Table:**

C_22_H_17_F_3_N_2_OS	*F*(000) = 856
*M**_r_* = 414.44	*D*_x_ = 1.401 Mg m^−^^3^
Orthorhombic, *P**c**a*2_1_	Mo *K*α radiation, λ = 0.71073 Å
Hall symbol: P 2c -2ac	Cell parameters from 5325 reflections
*a* = 20.0318 (4) Å	θ = 2.2–27.2°
*b* = 10.2866 (2) Å	µ = 0.21 mm^−^^1^
*c* = 9.5351 (2) Å	*T* = 100 K
*V* = 1964.79 (7) Å^3^	Plate, colourless
*Z* = 4	0.56 × 0.18 × 0.06 mm

### Data collection

**Table:**

Bruker SMART APEXII CCD area-detector diffractometer	5618 independent reflections
Radiation source: fine-focus sealed tube	4608 reflections with *I* > 2σ(*I*)
Graphite monochromator	*R*_int_ = 0.047
φ and ω scans	θ_max_ = 30.1°, θ_min_ = 2.0°
Absorption correction: multi-scan (*SADABS*; Bruker, 2009)	*h* = −27→28
*T*_min_ = 0.892, *T*_max_ = 0.987	*k* = −14→14
21265 measured reflections	*l* = −13→13

### Refinement

**Table:**

Refinement on *F*^2^	Secondary atom site location: difference Fourier map
Least-squares matrix: full	Hydrogen site location: inferred from neighbouring sites
*R*[*F*^2^ > 2σ(*F*^2^)] = 0.044	H atoms treated by a mixture of independent and constrained refinement
*wR*(*F*^2^) = 0.081	*w* = 1/[σ^2^(*F*_o_^2^) + (0.0299*P*)^2^ + 0.2655*P*] where *P* = (*F*_o_^2^ + 2*F*_c_^2^)/3
*S* = 1.02	(Δ/σ)_max_ = 0.001
5618 reflections	Δρ_max_ = 0.24 e Å^−^^3^
270 parameters	Δρ_min_ = −0.25 e Å^−^^3^
2 restraints	Absolute structure: Flack (1983), 2568 Freidel pairs
Primary atom site location: structure-invariant direct methods	Flack parameter: 0.01 (6)

### Special details

**Table:**

Experimental. The crystal was placed in the cold stream of an Oxford Cryosystems Cobra open-flow nitrogen cryostat (Cosier & Glazer, 1986) operating at 100.0 (1) K.
Geometry. All e.s.d.'s (except the e.s.d. in the dihedral angle between two l.s. planes) are estimated using the full covariance matrix. The cell e.s.d.'s are taken into account individually in the estimation of e.s.d.'s in distances, angles and torsion angles; correlations between e.s.d.'s in cell parameters are only used when they are defined by crystal symmetry. An approximate (isotropic) treatment of cell e.s.d.'s is used for estimating e.s.d.'s involving l.s. planes.
Refinement. Refinement of *F*^2^ against ALL reflections. The weighted *R*-factor *wR* and goodness of fit *S* are based on *F*^2^, conventional *R*-factors *R* are based on *F*, with *F* set to zero for negative *F*^2^. The threshold expression of *F*^2^ > σ(*F*^2^) is used only for calculating *R*-factors(gt) *etc*. and is not relevant to the choice of reflections for refinement. *R*-factors based on *F*^2^ are statistically about twice as large as those based on *F*, and *R*- factors based on ALL data will be even larger.

### Fractional atomic coordinates and isotropic or equivalent isotropic displacement parameters (Å^2^)

**Table:**

	*x*	*y*	*z*	*U*_iso_*/*U*_eq_	
F1	0.34230 (6)	0.45765 (11)	−0.02227 (17)	0.0418 (4)	
F2	0.32081 (5)	0.66244 (10)	−0.02775 (14)	0.0327 (3)	
F3	0.29424 (5)	0.54911 (10)	0.15416 (15)	0.0328 (3)	
S1	0.35402 (2)	0.73635 (4)	0.50740 (6)	0.02441 (11)	
N1	0.36053 (7)	0.78665 (14)	0.2330 (2)	0.0187 (3)	
N2	0.28048 (8)	0.90001 (14)	0.3579 (2)	0.0174 (3)	
O1	0.27383 (6)	0.95082 (11)	0.12731 (15)	0.0193 (3)	
C1	0.14450 (8)	1.10138 (16)	0.0592 (2)	0.0181 (4)	
H1A	0.1299	1.0144	0.0731	0.022*	
C2	0.12280 (9)	1.16913 (18)	−0.0574 (2)	0.0224 (4)	
H2A	0.0944	1.1282	−0.1238	0.027*	
C3	0.14277 (9)	1.29838 (18)	−0.0774 (2)	0.0257 (5)	
H3A	0.1276	1.3458	−0.1568	0.031*	
C4	0.18467 (8)	1.35616 (16)	0.0193 (2)	0.0243 (5)	
H4A	0.1982	1.4439	0.0065	0.029*	
C5	0.20734 (8)	1.28682 (16)	0.1358 (2)	0.0222 (4)	
H5A	0.2365	1.3272	0.2012	0.027*	
C6	0.18726 (8)	1.15807 (15)	0.1565 (2)	0.0168 (4)	
C7	0.20977 (8)	1.08378 (15)	0.2869 (2)	0.0152 (4)	
H7A	0.2368	1.1458	0.3444	0.018*	
C8	0.14922 (8)	1.04613 (16)	0.3756 (2)	0.0161 (4)	
C9	0.12615 (9)	1.13343 (17)	0.4758 (2)	0.0217 (4)	
H9A	0.1498	1.2121	0.4917	0.026*	
C10	0.06891 (9)	1.10700 (18)	0.5532 (2)	0.0253 (4)	
H10A	0.0533	1.1678	0.6206	0.030*	
C11	0.03473 (9)	0.99143 (19)	0.5314 (2)	0.0273 (5)	
H11A	−0.0043	0.9727	0.5843	0.033*	
C12	0.05759 (9)	0.90365 (18)	0.4329 (2)	0.0240 (5)	
H12A	0.0341	0.8246	0.4183	0.029*	
C13	0.11465 (9)	0.92994 (16)	0.3548 (2)	0.0201 (4)	
H13A	0.1301	0.8689	0.2874	0.024*	
C14	0.25639 (8)	0.97102 (14)	0.2475 (2)	0.0148 (3)	
C15	0.33231 (8)	0.80792 (16)	0.3580 (2)	0.0176 (4)	
C16	0.41583 (8)	0.70043 (16)	0.2104 (2)	0.0185 (4)	
C17	0.47773 (9)	0.72797 (17)	0.2680 (2)	0.0216 (4)	
H17A	0.4830	0.8010	0.3280	0.026*	
C18	0.53209 (9)	0.64879 (18)	0.2380 (2)	0.0260 (5)	
H18A	0.5745	0.6681	0.2774	0.031*	
C19	0.52477 (9)	0.54208 (18)	0.1511 (3)	0.0295 (5)	
H19A	0.5619	0.4874	0.1320	0.035*	
C20	0.46349 (9)	0.51515 (18)	0.0922 (3)	0.0269 (5)	
H20A	0.4586	0.4424	0.0317	0.032*	
C21	0.40852 (9)	0.59409 (16)	0.1208 (2)	0.0213 (4)	
C22	0.34224 (9)	0.56611 (17)	0.0566 (3)	0.0261 (5)	
H1N2	0.2679 (10)	0.9143 (17)	0.4368 (17)	0.015 (6)*	
H1N1	0.3439 (11)	0.823 (2)	0.147 (3)	0.049 (7)*	

### Atomic displacement parameters (Å^2^)

**Table:**

	*U*^11^	*U*^22^	*U*^33^	*U*^12^	*U*^13^	*U*^23^
F1	0.0392 (7)	0.0294 (6)	0.0567 (11)	−0.0032 (5)	−0.0016 (8)	−0.0226 (7)
F2	0.0323 (6)	0.0328 (6)	0.0330 (8)	−0.0032 (5)	−0.0084 (6)	−0.0004 (6)
F3	0.0265 (5)	0.0316 (6)	0.0403 (9)	−0.0078 (4)	0.0048 (6)	−0.0017 (6)
S1	0.0327 (2)	0.02260 (19)	0.0179 (2)	0.00898 (17)	0.0012 (3)	0.0045 (2)
N1	0.0213 (7)	0.0191 (7)	0.0156 (9)	0.0036 (6)	−0.0009 (8)	−0.0003 (7)
N2	0.0215 (7)	0.0200 (7)	0.0107 (9)	0.0040 (6)	0.0024 (8)	−0.0003 (7)
O1	0.0197 (6)	0.0248 (6)	0.0135 (8)	0.0022 (5)	−0.0008 (6)	0.0012 (6)
C1	0.0161 (7)	0.0196 (8)	0.0187 (10)	−0.0002 (6)	0.0020 (8)	0.0020 (8)
C2	0.0202 (8)	0.0273 (9)	0.0196 (11)	0.0014 (7)	−0.0015 (9)	0.0023 (9)
C3	0.0211 (9)	0.0286 (9)	0.0274 (13)	0.0056 (7)	0.0021 (9)	0.0119 (9)
C4	0.0224 (8)	0.0200 (7)	0.0306 (13)	0.0011 (6)	0.0028 (10)	0.0105 (9)
C5	0.0185 (8)	0.0188 (7)	0.0293 (13)	−0.0021 (6)	0.0018 (10)	0.0033 (9)
C6	0.0150 (7)	0.0169 (7)	0.0186 (11)	0.0018 (6)	0.0020 (8)	0.0023 (8)
C7	0.0163 (7)	0.0154 (7)	0.0138 (10)	−0.0015 (6)	−0.0004 (8)	−0.0005 (7)
C8	0.0165 (7)	0.0195 (8)	0.0124 (10)	0.0018 (6)	−0.0008 (8)	0.0021 (7)
C9	0.0228 (8)	0.0226 (7)	0.0196 (12)	0.0038 (6)	−0.0019 (9)	0.0013 (8)
C10	0.0252 (9)	0.0319 (9)	0.0188 (11)	0.0098 (7)	0.0021 (9)	0.0015 (9)
C11	0.0180 (8)	0.0378 (10)	0.0260 (14)	0.0047 (7)	0.0044 (10)	0.0123 (10)
C12	0.0190 (8)	0.0265 (9)	0.0266 (13)	−0.0021 (7)	−0.0007 (9)	0.0081 (9)
C13	0.0188 (8)	0.0209 (8)	0.0205 (11)	0.0003 (6)	−0.0009 (9)	0.0038 (9)
C14	0.0132 (7)	0.0164 (7)	0.0147 (10)	−0.0030 (6)	−0.0007 (8)	0.0026 (8)
C15	0.0199 (8)	0.0142 (7)	0.0187 (10)	−0.0012 (6)	0.0015 (9)	0.0011 (8)
C16	0.0195 (8)	0.0176 (7)	0.0184 (11)	0.0030 (6)	0.0024 (9)	0.0021 (8)
C17	0.0239 (9)	0.0209 (8)	0.0199 (11)	0.0006 (7)	−0.0005 (9)	0.0008 (8)
C18	0.0201 (8)	0.0277 (9)	0.0303 (13)	0.0017 (7)	0.0004 (10)	0.0047 (10)
C19	0.0255 (9)	0.0252 (9)	0.0378 (15)	0.0072 (7)	0.0066 (11)	0.0030 (10)
C20	0.0301 (9)	0.0196 (8)	0.0309 (14)	0.0026 (7)	0.0053 (10)	−0.0042 (9)
C21	0.0231 (8)	0.0162 (7)	0.0247 (12)	−0.0007 (6)	0.0029 (9)	−0.0006 (8)
C22	0.0275 (9)	0.0197 (8)	0.0313 (13)	−0.0021 (7)	0.0027 (10)	−0.0066 (9)

### Geometric parameters (Å, º)

**Table:**

F1—C22	1.345 (2)	C7—C14	1.536 (2)
F2—C22	1.346 (2)	C7—H7A	1.0000
F3—C22	1.349 (2)	C8—C9	1.391 (3)
S1—C15	1.661 (2)	C8—C13	1.395 (2)
N1—C15	1.337 (3)	C9—C10	1.390 (3)
N1—C16	1.435 (2)	C9—H9A	0.9500
N1—H1N1	0.96 (3)	C10—C11	1.388 (3)
N2—C14	1.369 (3)	C10—H10A	0.9500
N2—C15	1.405 (2)	C11—C12	1.382 (3)
N2—H1N2	0.807 (15)	C11—H11A	0.9500
O1—C14	1.216 (2)	C12—C13	1.391 (3)
C1—C2	1.382 (3)	C12—H12A	0.9500
C1—C6	1.391 (3)	C13—H13A	0.9500
C1—H1A	0.9500	C16—C17	1.386 (2)
C2—C3	1.402 (3)	C16—C21	1.395 (3)
C2—H2A	0.9500	C17—C18	1.390 (2)
C3—C4	1.382 (3)	C17—H17A	0.9500
C3—H3A	0.9500	C18—C19	1.383 (3)
C4—C5	1.396 (3)	C18—H18A	0.9500
C4—H4A	0.9500	C19—C20	1.378 (3)
C5—C6	1.398 (2)	C19—H19A	0.9500
C5—H5A	0.9500	C20—C21	1.395 (2)
C6—C7	1.527 (3)	C20—H20A	0.9500
C7—C8	1.529 (2)	C21—C22	1.490 (3)
			
C15—N1—C16	124.13 (18)	C12—C11—C10	119.86 (18)
C15—N1—H1N1	123.6 (14)	C12—C11—H11A	120.1
C16—N1—H1N1	112.2 (15)	C10—C11—H11A	120.1
C14—N2—C15	128.37 (19)	C11—C12—C13	120.64 (18)
C14—N2—H1N2	120.6 (14)	C11—C12—H12A	119.7
C15—N2—H1N2	110.6 (14)	C13—C12—H12A	119.7
C2—C1—C6	121.25 (16)	C12—C13—C8	119.89 (19)
C2—C1—H1A	119.4	C12—C13—H13A	120.1
C6—C1—H1A	119.4	C8—C13—H13A	120.1
C1—C2—C3	119.90 (18)	O1—C14—N2	122.15 (15)
C1—C2—H2A	120.1	O1—C14—C7	122.26 (17)
C3—C2—H2A	120.1	N2—C14—C7	115.46 (18)
C4—C3—C2	119.35 (19)	N1—C15—N2	114.95 (19)
C4—C3—H3A	120.3	N1—C15—S1	125.53 (14)
C2—C3—H3A	120.3	N2—C15—S1	119.53 (16)
C3—C4—C5	120.61 (16)	C17—C16—C21	119.80 (16)
C3—C4—H4A	119.7	C17—C16—N1	120.32 (16)
C5—C4—H4A	119.7	C21—C16—N1	119.68 (16)
C4—C5—C6	120.18 (18)	C16—C17—C18	119.99 (18)
C4—C5—H5A	119.9	C16—C17—H17A	120.0
C6—C5—H5A	119.9	C18—C17—H17A	120.0
C1—C6—C5	118.69 (17)	C19—C18—C17	120.36 (18)
C1—C6—C7	120.99 (14)	C19—C18—H18A	119.8
C5—C6—C7	120.27 (17)	C17—C18—H18A	119.8
C6—C7—C8	110.06 (13)	C20—C19—C18	119.87 (17)
C6—C7—C14	111.04 (16)	C20—C19—H19A	120.1
C8—C7—C14	115.23 (13)	C18—C19—H19A	120.1
C6—C7—H7A	106.7	C19—C20—C21	120.42 (18)
C8—C7—H7A	106.7	C19—C20—H20A	119.8
C14—C7—H7A	106.7	C21—C20—H20A	119.8
C9—C8—C13	119.07 (17)	C20—C21—C16	119.55 (17)
C9—C8—C7	118.72 (15)	C20—C21—C22	120.68 (17)
C13—C8—C7	122.14 (17)	C16—C21—C22	119.77 (16)
C10—C9—C8	120.82 (17)	F1—C22—F2	106.09 (19)
C10—C9—H9A	119.6	F1—C22—F3	106.16 (14)
C8—C9—H9A	119.6	F2—C22—F3	106.26 (15)
C11—C10—C9	119.71 (18)	F1—C22—C21	112.90 (15)
C11—C10—H10A	120.1	F2—C22—C21	112.79 (15)
C9—C10—H10A	120.1	F3—C22—C21	112.10 (19)
			
C6—C1—C2—C3	−1.4 (3)	C8—C7—C14—O1	128.74 (18)
C1—C2—C3—C4	0.7 (3)	C6—C7—C14—N2	178.66 (14)
C2—C3—C4—C5	0.3 (3)	C8—C7—C14—N2	−55.4 (2)
C3—C4—C5—C6	−0.6 (3)	C16—N1—C15—N2	177.55 (15)
C2—C1—C6—C5	1.1 (3)	C16—N1—C15—S1	−2.0 (3)
C2—C1—C6—C7	178.57 (16)	C14—N2—C15—N1	−1.1 (3)
C4—C5—C6—C1	−0.1 (3)	C14—N2—C15—S1	178.46 (14)
C4—C5—C6—C7	−177.58 (16)	C15—N1—C16—C17	−65.9 (2)
C1—C6—C7—C8	−60.0 (2)	C15—N1—C16—C21	119.3 (2)
C5—C6—C7—C8	117.41 (17)	C21—C16—C17—C18	−0.8 (3)
C1—C6—C7—C14	68.76 (19)	N1—C16—C17—C18	−175.70 (19)
C5—C6—C7—C14	−113.78 (17)	C16—C17—C18—C19	−0.2 (3)
C6—C7—C8—C9	−88.1 (2)	C17—C18—C19—C20	1.0 (3)
C14—C7—C8—C9	145.37 (17)	C18—C19—C20—C21	−0.7 (3)
C6—C7—C8—C13	88.9 (2)	C19—C20—C21—C16	−0.4 (3)
C14—C7—C8—C13	−37.6 (3)	C19—C20—C21—C22	179.5 (2)
C13—C8—C9—C10	−1.0 (3)	C17—C16—C21—C20	1.1 (3)
C7—C8—C9—C10	176.08 (17)	N1—C16—C21—C20	176.01 (19)
C8—C9—C10—C11	0.8 (3)	C17—C16—C21—C22	−178.75 (18)
C9—C10—C11—C12	−0.3 (3)	N1—C16—C21—C22	−3.9 (3)
C10—C11—C12—C13	0.0 (3)	C20—C21—C22—F1	2.8 (3)
C11—C12—C13—C8	−0.2 (3)	C16—C21—C22—F1	−177.32 (18)
C9—C8—C13—C12	0.7 (3)	C20—C21—C22—F2	−117.5 (2)
C7—C8—C13—C12	−176.30 (18)	C16—C21—C22—F2	62.4 (3)
C15—N2—C14—O1	8.3 (3)	C20—C21—C22—F3	122.6 (2)
C15—N2—C14—C7	−167.60 (16)	C16—C21—C22—F3	−57.5 (2)
C6—C7—C14—O1	2.8 (2)		

### Hydrogen-bond geometry (Å, º)

*Cg*1 is the centroid of the C1–C6 ring.

**Table:**

*D*—H···*A*	*D*—H	H···*A*	*D*···*A*	*D*—H···*A*
N1—H1*N*1···O1	0.96 (3)	1.93 (2)	2.6237 (19)	127 (2)
N2—H1*N*2···O1^i^	0.81 (2)	2.04 (2)	2.838 (2)	174 (2)
C9—H9*A*···F1^ii^	0.95	2.53	3.395 (2)	151
C7—H7*A*···*Cg*1^iii^	1.00	2.84	3.7826 (19)	158

Symmetry codes: (i) −*x*+1/2, *y*, *z*+1/2; (ii) −*x*+1/2, *y*+1, *z*+1/2; (iii) *x*+1/2, −*y*, *z*.
